# Effect of Chitosan–Riboflavin Bioconjugate on Green Mold Caused by *Penicillium digitatum* in Lemon Fruit

**DOI:** 10.3390/polym16070884

**Published:** 2024-03-23

**Authors:** Brenda M. Ipinza-Concha, Luciano Dibona-Villanueva, Denis Fuentealba, Alexander Pinilla-Quispe, Daniel Schwantes, María A. Garzón-Nivia, Mario A. Herrera-Défaz, Héctor A. Valdés-Gómez

**Affiliations:** 1 Facultad de Agronomía y Sistemas Naturales, Pontificia Universidad Católica de Chile, Santiago 7820436, Chile; bmipinza@uc.cl (B.M.I.-C.);; 2 Facultad de Química y de Farmacia, Pontificia Universidad Católica de Chile, Santiago 7820436, Chile; 3 Facultad de Medicina, Pontificia Universidad Católica de Chile, Santiago 7820436, Chile

**Keywords:** antifungal, *Penicillium digitatum*, biofungicide, chitosan, riboflavin, photoactive, citrus

## Abstract

*Penicillium digitatum* is the causal agent of green mold, a primary postharvest disease of citrus fruits. This study evaluated the efficacy of a novel photoactive chitosan–riboflavin bioconjugate (CH-RF) to control green mold in vitro and in lemon fruit. The results showed total inhibition of *P. digitatum* growth on APDA supplemented with CH-RF at 0.5% (*w*/*v*) and a significant reduction of 84.8% at 0.25% (*w*/*v*). Lemons treated with CH-RF and kept under controlled conditions (20 °C and 90–95% relative humidity) exhibited a noteworthy reduction in green mold incidence four days post-inoculation. Notably, these effects persisted, with all treatments remaining significantly distinct from the control group until day 14. Furthermore, CH-RF showed high control of green mold in lemons after 20 days of cold storage (5 ± 1 °C). The disease incidence five days after cold storage indicated significant differences from the values observed in the control. Most CH-RF treatments showed enhanced control of green mold when riboflavin was activated by white-light exposure. These findings suggest that this novel fungicide could be a viable alternative to conventional synthetic fungicides, allowing more sustainable management of lemon fruit diseases.

## 1. Introduction

*Penicillium digitatum* is a phytopathogenic fungus of the Ascomycota phylum that causes green mold in citrus fruits. Along with *Penicillium italicum* (blue mold), these are the primary sources of decay loss in citrus production worldwide [1]. *P. digitatum* is a pathogen that infects fruit through wounds that can occur accidentally in the field during handling, due to insects or thorns, or in transportation [2]. This pathogen is primarily controlled by synthetic fungicides used before cold storage [3], which are applied as a wax coating, dip, or drench [4]. Despite their extensive use, conventional fungicides are under critical scrutiny due to their adverse environmental effects and potential risks to human health. These effects include the contamination of surface water sources, leading to toxicity to different species, such as invertebrates, vertebrates, and microorganisms [5]. In terms of human health, there is evidence of toxic effects on human cells [6] and neurological disruption in neonates [7]. Furthermore, the extensive and frequent use of fungicides has led to the emergence of resistant strains of many *Penicillium* species [8]. These factors have promoted research into alternative biodegradable and less toxic fungicides.

Chitosan is the deacetylated form of chitin, one of the most abundant polymers in nature. It is a constituent of the exoskeleton of crustaceans and insects, but it can also be found as a component of fungal cells [9]. Chitosan has been extensively studied due to its antifungal and resistance-inducing properties. In this context, assays have been conducted under preharvest and postharvest conditions to control fungal decay in fruits [10]. Chitosan alone, associated or in combination with other compounds, has been tested on postharvest fungi such as *Botrytis cinerea* [11,12], *Colletotrichum gloeosporioides* [13], and *Penicillium italicum* [14]. Chitosan has also been tested to control *P. digitatum* decay in several citrus species, with variable results. In grapefruit, a reduction in severity was observed, but no reduction in incidence six days after inoculation [15]. In assays in mandarins, a reduction in incidence and severity was observed seven days after inoculation [16]. Similar results were observed on cold storage [17], while other authors report no reduction in incidence four days after inoculation [18].

Photodynamic inactivation (PDI) is a promising alternative method that has been studied due to its capacity to treat human diseases. This technique involves the use of a photosensitizer at a specific wavelength of light in the presence of oxygen to generate reactive oxygen compounds that trigger cell damage and death [19]. PDI has recently been tested for the inactivation of human pathogenic fungal species such as *Trichophyton rubrum*, *Candida albicans*, and Tinea [20]. Studies on the control of phytopathogenic fungi are very limited. Some of the photosensitizers studied in plant disease control include curcumin for *B. cinerea* [21] and *P. expansum* [22], harmol for *P. digitatum* and *B. cinerea* [23], and vitamin K3 in *P. digitatum* [24].

In this context, a cutting-edge photoactive fungicide crafted from the combination of chitosan and riboflavin (vitamin B2) has been developed [25]. The fungicidal effect of this molecule (CH-RF) is driven by exposure to white light. This molecule has been shown to significantly inhibit the growth of *B. cinerea* in a culture medium and significantly reduce its incidence in inoculated grapes in a concentration-dependent manner [12]. Preliminary results have shown that CH-RF inhibits the mycelial growth of *P. digitatum* at low doses under in vitro conditions [25,26]. Nevertheless, its efficacy in preventing *P. digitatum* infection in citrus fruits and its performance under postharvest conditions remain unexplored.

Thus, this study aims to evaluate the efficacy of the photoactive molecule composed of chitosan–riboflavin (CH-RF) in controlling the damage caused by *Penicillium digitatum*. Efficacy was assessed under three conditions: in vitro in Petri dishes in laboratory biotests, in lemon fruits under a high-susceptibility environment (20 °C; 90–95% RH), and in microscale cold storage followed by shelf-life postharvest conditions.

## 2. Materials and Methods

### 2.1. Plant Material

Commercially mature lemons (*Citrus limon* L. Burm. f. cv. Eureka frost) without recent fungicide applications were harvested from an orchard in the Melipilla area, Chile. The absence of recent fungicide applications ensures the conditions for green mold growth. Fruits were selected based on uniformity, ripeness, and absence of mechanical damage.

### 2.2. Fungal Isolates

The *Penicillium digitatum* strain used in the experiments was obtained from the Fruit Pathology Laboratory of the Agricultural and Natural Systems Faculty of the Pontificia Universidad Católica de Chile. This pathogen was obtained from diseased fruits and maintained in a 20% glycerol solution at −20 °C until use. Conidial suspension for the different experiments was obtained from 7-day-old *P. digitatum* growth on acidified potato dextrose agar (APDA) at 20 ± 1 °C. A 1 × 1 cm portion of the recent sporulated fungal growth was scraped from the culture medium and mixed with 0.05% (*v*/*v*) Tween 80 detergent to prepare the pathogen suspension. This solution was poured into a glass beaker through a sterilized double-gauze layer to remove the mycelia. An aliquot of 5 µL of the suspension was extracted and placed on a hemocytometer (BOECO, Hamburg, Germany) to count the conidia under a light microscope (Olympus CX31, Tokyo, Japan). Concentration was adjusted using Tween 80^®^ solution. For in vitro assays, the suspension was adjusted to 1 × 10^5^ conidia mL^−1^; for the in vivo assays, the concentration was 2 × 10^5^ conidia mL^−1^ [13,17].

### 2.3. CH-RF Biofungicide

**Figure 1:**
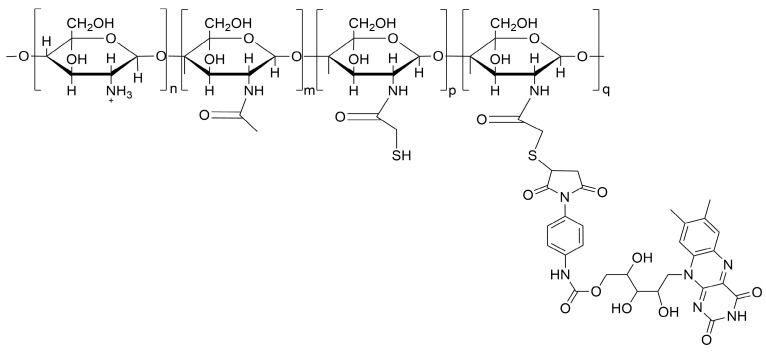
CH-RF chemical structure. The subscripts n, m, p and q depict the recurrent unit composition of free amino, acetylated, thiolated and RF containing glucosamine groups, respectively. Substitution degree n = 70%, m = 14%, p = 15% and q = 1%.

CH-RF biofungicide was provided by the Supramolecular Chemistry and Photobiology Laboratory of the Chemistry and Pharmacy Faculty of the Pontificia Universidad Católica de Chile. The protocol developed by Dibona-Villanueva and Fuentealba (2021) was implemented to elaborate the conjugate. Briefly, 2 mg of *N*-hydroxysuccinimide (NHS), 1 mL of thioglycolic acid, and 3.5 mg of 1-ethyl-3-(3-dimethylaminopropyl) carbodiimide (EDC) were dissolved in 2 mL of dimethylformamide (DMF) and stirred overnight at room temperature. Simultaneously, a 2.5% (*w*/*v*) chitosan solution (90% degree of deacetylation, ≤3.0 kDa, Chitolytic, Toronto, ON, Canada) was prepared in HCl 0.2 M and adjusted to pH 5 by adding 10 M NaOH. The activated NHS-ester obtained in the previous step was added to the chitosan solution and stirred for 24 h at room temperature. The thiolated chitosan (CH-SH) was recovered through dialysis separation, lyophilized, and stored in a cold and dark environment. A total of 500 mg of CH-SH was dissolved in 20 mL of water and adjusted to pH 6. Simultaneously, 19 mg of RF-PMPI previously synthetized using the protocols depicted by Dibona-Villanueva and Fuentealba (2021) was dissolved in a small amount of DMSO and added to the thiolated chitosan solution under constant stirring. The CH-SH-and-RF-PMPI mixture was stirred at room temperature under a N_2_ atmosphere and protected from light overnight. The CH-RF conjugate was recovered by ethanol precipitation and washed several times with ethanol and cold water until no color was observed in the filtrate. The product was then dried and stored at 4 °C while protected from light. The conjugate was characterized by the FTIR and MS techniques detailed in the previous report. The final structure of the conjugate as well as the percentual components are depicted in Figure 1.

### 2.4. In Vitro Tests

CH-RF’s efficacy in controling *P. digitatum* growth was assessed by a five-treatment in vitro assay. Each treatment was mixed with APDA containing 3.2 g potato puree, 3.2 g dextrose, 4 g agar–agar, and 80 μL lactic acid for a 160 mL solution. APDA was diluted in 160 mL of distilled water for control treatment and autoclaved at 121 °C for 15 min. For fludioxonil treatment (FLU 0.1), 160 μL of commercial fungicide Shield Brite FDL 230SC (Anasac Chile S.A, Lampa, Chile) was mixed with the 160 mL autoclaved APDA to reach a concentration of 0.1% (*v*/*v*) (0.023% *w*/*v* fludioxonil). APDA was mixed with 80 mL of a 1% (*w*/*v*) stock CH-RF solution to obtain a concentration of 0.5% (*v*/*v*) (CH-RF0.5). The same procedure was used to prepare CH-RF0.25 and CH-RF0.1. pH of CH-RF treatments ranged between 4.3 and 4.5. A total of 12 mL of the different culture media was poured into Petri dishes and left to solidify. An amount of 5 μL of conidial suspension of *P. digitatum* was placed at the center of each Petri dish. Each treatment consisted of five replicates of three plates. The treatments were maintained at 20 ± 1 °C under a light/dark cycle of 12:12 h.

### 2.5. Biotests in Lemon Fruit in Temperate and Cold Storage

Fruits were superficially disinfected with 2% *v*/*v* sodium hypochlorite solution for 2–3 min and washed twice with sterilized distilled water. Subsequently, lemons were air-dried in a laminar flow cabinet for 1–2 h. Lemons were wounded at the equatorial region using a scalpel, creating 2 × 2 mm lesions in depth and length. An aliquot of 10 μL of a *P. digitatum* conidial suspension (2 × 10^5^ conidia mL^−1^) was placed in the wound and left to dry for 5 h. Treatments were performed by dipping lemons in sterile water (control), Fludioxonil (FLU) 0.2% (*v*/*v*) (Shield Brite FDL 230SC, Anasac Chile S.A, Lampa, Chile), and CH-RF 2% (*w*/*v*) exposed for 0 min (CH-RF), 6 min (CH-RF6), 15 min (CH-RF15), and 30 min (Ch-RF30) to white LED with irradiance of 17–23 W/m^2^, and continuously rotated to illuminate the whole lemon surface. The control and FLU treatment groups were not exposed to light. An illumination step was required to activate riboflavin in the conjugate. Fruits were left to dry completely in a laminar flow cabinet for 1–2 h.

For temperate- and humid-condition assays, individual lemons that were inoculated and treated as indicated above were placed inside a sealed plastic container with a height of 15 cm and a diameter of 10 cm. The humidity level in the box was maintained at 90–95% by pouring 12 mL of sterilized distilled water into a paper towel at the bottom of the box. The fruit was placed above a rack and was not in direct contact with the wet bottom of the box. Plastic containers were stored at 20 ± 1 °C. Each treatment had four replicates of three boxes, with one lemon each.

For cold storage, lemons were arranged in cardboard packaging with fruit trays inside cardboard boxes to simulate the postharvest conditions of citrus fruits. Lemons were stored for 20 days at 5 ± 1 °C, and then, exposed to room temperature (20 ± 1 °C) for 9 days. The same treatments used for the humid box assays were implemented, and each treatment had four replicates of 16 fruits each. Experiments with lemon fruits in humid boxes and cold storage conditions were conducted twice.

### 2.6. Disease Evaluation

For the in vitro tests, the diameter of mycelium growth (mm) was measured daily until day 10, and the mean of the cross-measurements of the colony was calculated. Mycelial growth inhibition was calculated using the following equation:

MGI (%) = ((Dc − Dt)/Dc) × 100(1)

where MGI is mycelial growth inhibition, Dc is the diameter of mycelial growth in the control (mm), and Dt is the diameter of the treatment.

For both in vivo assays, the incidence (lemons with evident green mold infection) was calculated as the mean of the diseased lemons in the four replicates. The severity was assessed as the percentage of lemon surface with green mold growth [27]. This methodology was used to avoid cross-infection for manipulation and because of the round surface of the lemons. A lemon with half of its surface exhibiting green mold growth was estimated to have a severity of 50%. Efficacy in controlling the disease was evaluated using the following equation:

Efficacy (%) = ((Ci − Ti)/Ci) × 100(2)

where Ci is the incidence of the control, and Ti is the incidence of the treatment in evaluation.

Evaluation of lemons infected with green mold in humid boxes was carried out 4, 7, 11, and 14 days after inoculation. For the postharvest simulation tests, green mold infection was evaluated during cold storage on days 7 and 15 and at room temperature on days 0, 5, 7, and 9.

### 2.7. Statistical Analysis

Disease development in the different assays was analyzed using an ANOVA (Infostat 2020 version statistical software, Córdoba, Argentina). When significant effects were observed, differences between the means were determined using Tukey’s test (HSD) (*p* < 0.05). Data as percentages were arcsine angular transformed before running the analyses.

## 3. Results

### 3.1. In Vitro Performance of CH-RF

**Figure 2:**
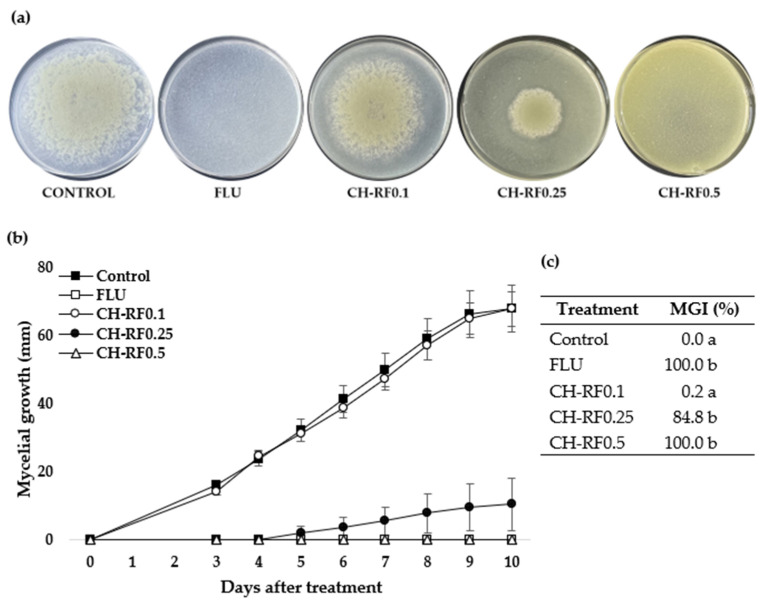
Effect of chitosan–riboflavin conjugate (CH-RF) on in vitro mycelial growth of *Penicillium digitatum*. (**a**) Growth of *P. digitatum* in the different treatments on day 10 post-sowing. (**b**) Mycelial growth (mm) from days 0 to 10 and (**c**) mycelial growth inhibition (MGI) (%) on day 10. Vertical bars represent the standard error of the mean. Different letters indicate statistical differences according to Tukey’s test (*p* < 0.05).

The results showed inhibition of *P. digitatum* growth in most CH-RF treatments (Figure 2). Green mold mycelia were observed on day 3 of evaluation for the control and CH-RF0.1 and on day 5 in the CH-RF0.25 treatment (Figure 2b). On day 10 of evaluation, there was no visible growth of *P. digitatum* on the CH-RF0.5 and FLU treatments (Figure 2a,b), while the control and CH-RF0.1 showed 68 mm and 67.8 mm growth, respectively. Mycelial growth was completely inhibited on the CH-RF0.5 and FLU treatments, while CH-RF0.25 showed a value of 84.5%. Non-significant differences (*p* < 0.05) were observed between the control and CH-RF0.1 (Figure 2c).

### 3.2. In Vivo Performance of CH-RF on Lemon Fruit in Temperate Condition

**Figure 3:**
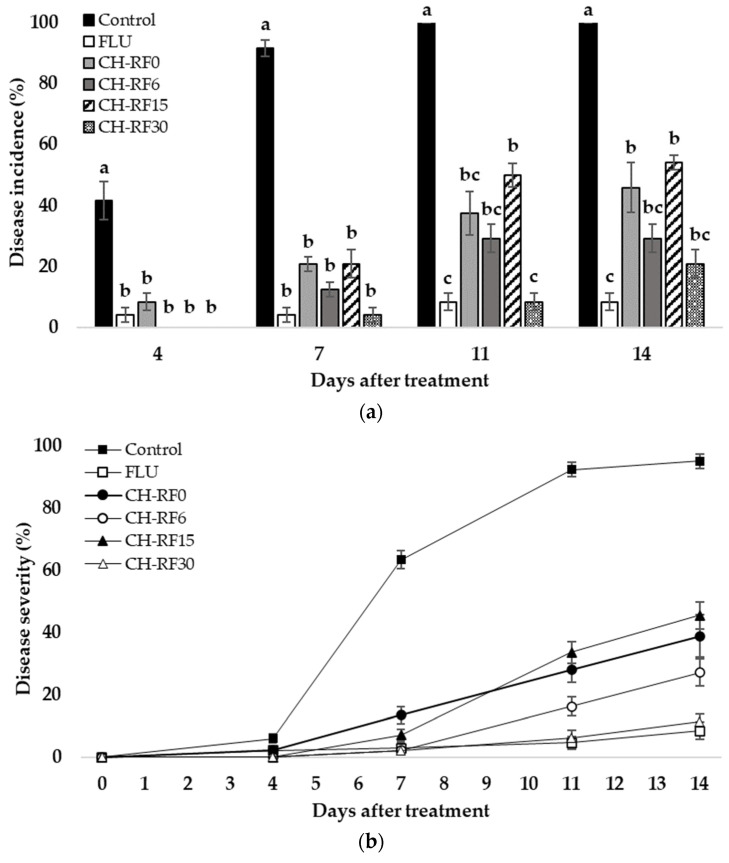

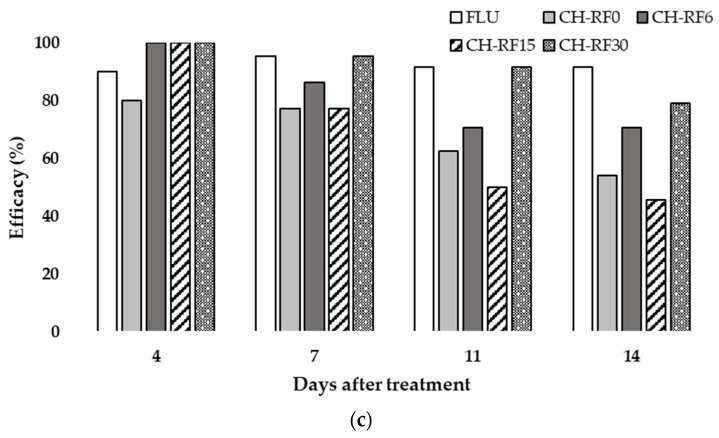
Effect of chitosan–riboflavin conjugate (CH-RF) on incidence (%) (**a**), mean severity (%) (**b**), and efficacy of control (%) (**c**) of green mold disease in lemon fruit inoculated, treated, and evaluated after 4, 7, 11, and 14 days in 20 ± 1 °C and 90–95% RH. Treatments consisted of sterile distilled water (control), fludioxonil 0.2% (FLU), or CH-RF 2% without (CH-RF0) or with 6 (CH-RF6), 15 (CH-RF15), and 30 min (CH-RF30) of white LED light exposure. The experiments were conducted twice. Vertical bars indicate standard errors of the mean. Different letters indicate statistical differences according to Tukey’s test (*p* < 0.05).

The incidence and severity of green mold were evaluated in lemons under optimal growth conditions for the pathogen (20 ± 1 °C 90–95% RH). Previous studies using CH-RF in lemons showed a lower efficacy in the control of green mold at concentrations of 0.7% and 1.4% (Supplementary Figures S1 and S2). It was then decided to use a concentration of 2% *w*/*v* of CH-FR. At this concentration, a significant reduction in green mold incidence was observed in inoculated lemons across all treatments compared to the control. Statistical differences were observed on days 4, 7, 11, and 14 post-inoculations (Figure 3a). All treatments showed an incidence below 8.5% and 21% for days 4 and 7, respectively, while the control incidence was 42% and 92% for the same days. No differences between the FLU and CH-RF treatments were observed for days 4 and 7. After that, all treatments remained significantly different from the control, but the lowest incidence was observed for FLU, CH-RF6, and CH-RF30 until day 14 of evaluation.

Disease severity was also evaluated in lemons on days 4, 7, 11, and 14 (Figure 3b), and their trends closely mirrored those observed for the incidence. A low severity (<6%) was observed in all treatments on day 4. On day 7, all treatments showed green mold growth, with the control exhibiting an increase in severity, reaching 63.3%, followed by CH-RF0 at 13.4%. On day 11, the control displayed a disease severity of 92.3%, followed by CH-RF15 with 33.5%. On the last evaluation day, the control reached 95% severity, and CH-RF15 reached 45.4%. In this assessment, all treatments demonstrated statistical differences from the control; however, the treatments with the lowest severity values were consistently observed in FLU, CH-RF6, and CH-RF30.

All treatments showed substantial efficacy in the control of green mold (Figure 3c). Treatments CH-RF6, CH-RF15, and CH-RF30 showed 100% efficacy on day 4 of the evaluation. FLU remained the most effective treatment until day 14 (92%), followed by CH-RF30 (79%), and CH-RF6 (71%).

### 3.3. In Vivo Performance of CH-RF in Lemon Fruit in Cold Storage

**Figure 4:**
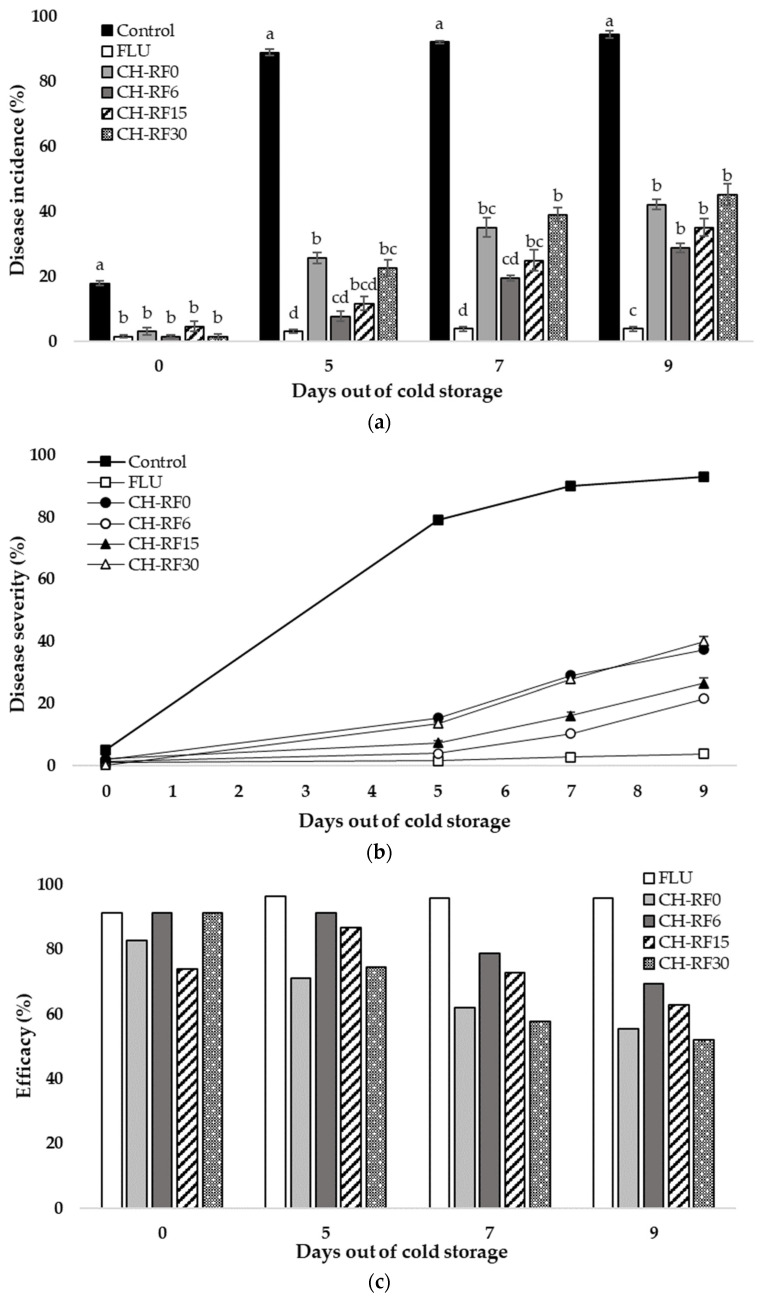
Effect of chitosan–riboflavin conjugate (CH-RF) on incidence (**a**), mean severity (**b**), and efficacy of control (**c**) of green mold disease in lemon fruit inoculated, treated, and maintained in cold storage for 14 days and evaluated after 0, 5, 7, and 9 days at room temperature Treatments consisted of sterile distilled water (control), fludioxonil 0.2% (FLU), or CH-RF 2% without (CH-RF0) or with 6 (CH-RF6), 15 (CH-RF15), and 30 min (CH-RF30) of white LED light exposure. The experiments were conducted twice. Vertical bars indicate standard errors of the mean. Different letters indicate statistical differences according to Tukey’s test (*p* < 0.05).

Inoculated lemons were treated and kept for 20 days in cold storage (5 ± 1 °C), and then, placed at room temperature (20 ± 1 °C). Evaluations were made on days 0, 5, 7, and 9. The disease incidence results showed effective control of CH-RF over green mold throughout all evaluation days (Figure 4a). After 14 days of cold storage, the highest level of incidence was observed in the control, with 17.9% of lemons exhibiting green mold. On the fifth day post-release from cold storage, a notable increase in the occurrence of moldy fruits was observed. All CH-RF treatments differed from the control (89.1%), and the lowest levels of diseased lemons were observed in FLU, CH-RF6, and CH-RF15. Day 7 followed the trend of day 5, with FLU and CH-RF6 showing the best performance, with disease incidences of 3.9% and 19.5%, respectively. On day 9 of evaluation, the control showed a 94.5% incidence, while all CH-RF treatments were significantly different, with an incidence of 42.2% or below. In addition, all CH-RF treatments were different from FLU treatments.

The control reached 92.9% severity on day 9 of evaluation (Figure 4b), the highest value compared to the other treatments. The lowest severity was measured in FLU at 3.8% and CH-RF6 at 21.7%, both showing differences from the rest of the treatments.

The efficacy in controlling green mold observed in the cold storage tests, followed by subsequent maintenance at room temperature, was similar to that in the tests conducted in temperate temperatures and at high RH, showing a decline from day 0 to day 9 (Figure 4c). The conventional commercial fungicide FLU achieved 95.9% efficacy. Greater efficacy of the CH-RF treatments was observed for CH-RF6 (69.4%).

## 4. Discussion

Chitosan is a natural biopolymer with proven antifungal effects against various fungi. The control of green mold with chitosan has been previously evaluated in vitro and in different citrus species, such as lemons, oranges, and grapefruit [16,17,18,28,29]. In addition, there are increasing studies using photoactive compounds to inhibit or reduce the growth of several postharvest fungi. Examples include harmol [23] and vitamin K3 [24], both exhibiting promising potential control of *P. digitatum*. In this study, we evaluated the fungicidal effect of CH-RF, a photoactive conjugate made up of riboflavin (1%) and chitosan (99%), on *P. digitatum* in vitro tests and using infected lemon fruits. As demonstrated in the in vitro evaluations, the growth of *P. digitatum* was completely decreased by 0.5% *w*/*v* CH-RF. This trend is consistent with previous studies showing that chitosan at a concentration of 0.5% inhibits entirely or reduces the growth of the fungus by more than 90% [16]. Our findings reveal a heightened inhibitory impact resulting from the incorporation of riboflavin into chitosan. Notably, a 0.25% concentration of CH-RF effectively regulates the growth of *P. digitatum*, underscoring the substantial inhibitory potential of this composite. While other studies have demonstrated nearly complete control of pathogen growth using 0.25% chitosan alone, it is important to note that these investigations employed significantly lower spore concentrations compared to those utilized in our study [29].

*Efficacy of CH-RF on lemons under optimal growth conditions for the pathogen (20 °C; 90–95%RH)*: The in vivo experiments demonstrated the need to increase the doses that were effective in controlling the pathogen under in vitro conditions. The preliminary experiments with CH-RF in lemons in humid boxes showed lower antifungal effects at concentrations of 0.7% *w*/*v* and 1.4% *w*/*v*, with an efficacy of nearly 100% on day 5, which decreased on day 7 of evaluation (see Supplementary Materials). These results reaffirmed the use of 2% *w*/*v* CH-RF as the dose that showed a more extended inhibitory period. This result is consistent with previous reports about the use of chitosan in grapefruits, demonstrating nearly no control of the pathogen with 1% *w*/*v* chitosan [15] and no significant differences in the incidence on day 4 of evaluation compared to the control using chitosan even at 3% *w*/*v* [18]. Our results showed an increased and significant inhibitory effect on incidence and severity using 2% *w*/*v* CH-RF. The enhanced effect could be explained by the activation of the riboflavin portion of the conjugate. We can observe this effect by comparing the reduction in pathogen damage at seven days by chitosan alone with that of the CH-RF molecule (see Supplementary Materials).

*Efficacy of CH-RF under cold storage and shelf display conditions:* Cold storage is a commonly used method that extends the commercial life of citrus fruits and reduces the growth of several fungus species [30]. For this reason, the use of CH-RF for lemons, and the simulation of commercial cold storage and shelf-life conditions, were tested. Studies on mandarins treated with 1% *w*/*v* chitosan for 17 days in cold storage reported a 40% decay incidence due to *P. digitatum* [17]. Other reports using a coating of chitosan solution conducted with oranges showed no incidence of *P. digitatum* in cold storage for up to three or four weeks [29]. Also, using 2% *w*/*v* chitosan coating on mandarins, and then, storing them for 15 days at 5 °C and 5 days at 25 °C [18] found no reduction in the incidence of green mold but a reduction in decay severity. The differences across studies regarding cultivar susceptibility, inoculum concentration, or strain virulence may account for the inconsistent results. In our study, green mold was scarcely present in the evaluations during the 20 days of cold storage. Disease incidence was observed only when the fruits were exposed to temperate temperature conditions (day 0). On day 5, it was below 25% for all treatments with CH-RF and light exposure. This difference could be explained by the enhanced fungicidal mechanism shown by the riboflavin portion of the conjugate, suggesting greater performance of the CH-RF conjugate above CH by itself.

*Influence of light exposure on CH-RF efficacy*: Li et al. [24] observed increased inhibition of the spore germination of a photoactive vitamin K3 analog when exposed to UV and sunlight compared to its use in the dark. Dibona-Villanueva and Fuentealba [25] conducted pioneering in vitro experiments using CH-RF at a 0.5% *w*/*v* concentration. Their findings revealed the remarkable complete inhibition of *P. digitatum* growth under visible light irradiation, showcasing the powerful antifungal effect of CH-RF. In contrast, the inhibition rate dropped to approximately 25% when the experiment was conducted in dark conditions, underscoring the crucial role of light in enhancing the inhibitory efficacy against *P. digitatum*. In our experiments, we consistently observe enhanced efficacy in pathogen control when the CH-RF molecule is irradiated for 6 min, as opposed to situations where no initial irradiation is applied. For alternative irradiation durations (15 or 30 min), an improvement in control is not consistently observed. The observed variations in the latter results can be attributed to the following: (i) prolonged LED light exposure, resulting in faster riboflavin photodecomposition [31], and consequently, the efficacy diminishes over time; (ii) in the fruits where CH-RF was applied without initial LED light exposure, they were kept in a natural-light environment in the laboratory, rather than complete darkness. This result suggests the possibility of some activation of riboflavin taking place. Nonetheless, it has to be mentioned that the trend shown in Figure 4 of large reductions at low light exposure and a reduced effect at higher light exposure aligns with the general trends in the literature [32]. The effect of delivering a fixed amount of energy through either a low-power source over extended periods or a high-power source over shorter durations and the biological implications of both remain a topic of ongoing inquiry.

*Potential mechanisms explaining CH-RF efficacy:* The precise mechanisms responsible for CH-RF’s antifungal activity remain incompletely elucidated; however, several potential pathways have been suggested. Previous studies have shed light on potential pathways for the effectiveness of photodynamic inactivation (PDI), particularly in disrupting microbial cell membranes [33]. Furthermore, the critical role of reactive oxygen species (ROS) in causing oxidative stress within the cell has been highlighted [34]. Notably, the involvement of ergosterol degradation in the PDI mechanism has been a focal point of recent investigations. Ergosterol, a vital component of fungal cell membranes, is identified as a target during PDI, with studies demonstrating its degradation as a crucial step in the photodynamic inactivation process [35]. Dibona-Villanueva and Fuentealba [25,36] described the importance of the localization and accumulation of photosensitizers in fungal spores’ superficial cell structures in photoinactivation extent. Additionally, there is evidence that supports the idea that the photoinactivation effect strongly depends on the interaction of photosensitizers and fungal cell envelopes [37].

These findings deepen our comprehension of the mechanisms underlying the efficacy of the chitosan–riboflavin bioconjugate, providing valuable insights for fine-tuning protocols and applications against a range of fungal pathogens. Furthermore, these results suggest that this novel fungicide could be used as a promising alternative to traditional synthetic fungicides, enabling a more sustainable approach to the management of lemon fruit diseases.

## 5. Conclusions

The present study demonstrated the antifungal effect of a novel chitosan–riboflavin bioconjugate (CH-RF) on the growth of *P. digitatum*, one of the most relevant postharvest pathogens in citrus fruit. The results indicate better performance of the bioconjugate compared to the use of chitosan by itself. Moreover, most CH-RF treatments showed enhanced control of green mold when riboflavin was activated by white-light exposure. Our study suggests that this conjugate could be an excellent natural fungicide for the biological control of green mold and could thus be used as an alternative to synthetic fungicides. Further investigations focused on evaluating the bioconjugate in different pathogens and fruit species are currently being pursued.

## 6. Patents

Patent application PCT/CL2020/050154 “photoactive biofungicide” USA: No. 17/784,797 (13 June 2022). European Patent Office 20899708.0 (11 July 2022). Brazil: No. BR 11 2022 011326 (9 June 2022). Patent application INAPI No: 201903654, Chile.

## Data Availability

Data will be made available upon reasonable request.

## Supplementary Materials

The following supporting information can be downloaded at: https://www.mdpi.com/article/10.3390/polym16070884/s1, Figure S1: Disease incidence (%) of green mold on lemons inoculated and treated with sterile water (control); chitosan–riboflavin conjugate at 0.7% with 15 (CH-RF0.7% 15) and 30 min (CH-RF0.7% 30) of LED exposure or CH-RF at 2% with 15 (CH-RF2% 15) and 30 min (CH-RF2% 30) of LED exposure; Figure S2: Disease incidence (%) of green mold on lemons inoculated and treated with sterile water without (control0) and with 15 min of white LED exposure (control15); chitosan at 1.4% (CH1.4%); chitosan–riboflavin conjugate at 0.7% with 30 min of LED exposure (CH-RF0.7% 30) and CH-RF at 1.4% with 15 min of LED exposure (CH-RF1.4% 15). Figure S3: Disease severity (%) of green mold on lemons inoculated and treated with sterile water (Control); Chitosan-Riboflavin conjugate at 0.7% with 15 (CH-RF0.7% 15) and 30 min (CH-RF0.7% 30) of LED exposure or CH-RF at 2% with 15 (CH-RF2% 15) and 30 min (CH-RF2% 30) of LED. Figure S4: Disease severity (%) of green mold on lemons inoculated and treated with sterile water without (Control0) and with 15 min of white LED exposure (Control15); Chitosan at 1.4% (CH1.4%); Chitosan-Riboflavin conjugate at 0.7% with 30 min of LED exposure (CH-RF0.7% 30) and CH-RF at 1.4% with 15 min of LED exposure (CH-RF1.4% 15).

## References

[1] Costa J.H., Bazioli J.M., de Moraes Pontes J.G., Fill T.P. (2019). Penicillium Digitatum Infection Mechanisms in Citrus: What Do We Know so Far?. Fungal Biol..

[2] Palou L. (2014). Penicillium Digitatum, Penicillium Italicum (Green Mold, Blue Mold). Postharvest Decay.

[3] Ladaniya M. (2023). Postharvest Disease Management with Fungicides. Citrus Fruit.

[4] Kellerman M., Liebenberg E., Njombolwana N., Erasmus A., Fourie P.H. (2018). Postharvest Dip, Drench and Wax Coating Application of Pyrimethanil on Citrus Fruit: Residue Loading and Green Mould Control. Crop Prot..

[5] Zubrod J.P., Bundschuh M., Arts G., Brühl C.A., Imfeld G., Knäbel A., Payraudeau S., Rasmussen J.J., Rohr J., Scharmüller A. (2019). Fungicides: An Overlooked Pesticide Class?. Environ. Sci. Technol..

[6] Xu J., Xiong H., Zhang X., Muhayimana S., Liu X., Xue Y., Huang Q. (2020). Comparative Cytotoxic Effects of Five Commonly Used Triazole Alcohol Fungicides on Human Cells of Different Tissue Types. J. Environ. Sci. Health Part B.

[7] Wang Y., Lafon P.-A., Salvador-Prince L., Gines A.R., Trousse F., Torrent J., Prevostel C., Crozet C., Liu J., Perrier V. (2021). Prenatal Exposure to Low Doses of Fungicides Corrupts Neurogenesis in Neonates. Environ. Res..

[8] Oiki S., Yaguchi T., Urayama S., Hagiwara D. (2022). Wide Distribution of Resistance to the Fungicides Fludioxonil and Iprodione in Penicillium Species. PLoS ONE.

[9] Muanprasat C., Chatsudthipong V. (2017). Chitosan Oligosaccharide: Biological Activities and Potential Therapeutic Applications. Pharmacol. Ther..

[10] Romanazzi G., Feliziani E., Baños S.B., Sivakumar D. (2017). Shelf Life Extension of Fresh Fruit and Vegetables by Chitosan Treatment. Crit. Rev. Food Sci. Nutr..

[11] Hua C., Li Y., Wang X., Kai K., Su M., Shi W., Zhang D., Liu Y. (2019). The Effect of Low and High Molecular Weight Chitosan on the Control of Gray Mold (*Botrytis Cinerea*) on Kiwifruit and Host Response. Sci. Hortic..

[12] Herrera-Défaz M., Fuentealba D., Dibona-Villanueva L., Schwantes D., Jiménez B., Ipinza B., Latorre B., Valdés-Gómez H., Fermaud M. (2023). Biocontrol of Botrytis Cinerea on Grape Berries in Chile: Use of Registered Biofungicides and a New Chitosan-Based Fungicide. Horticulturae.

[13] Zhao Y., Deng L., Zhou Y., Ming J., Yao S., Zeng K. (2018). Wound Healing in Citrus Fruit Is Promoted by Chitosan and Pichia Membranaefaciens as a Resistance Mechanism against Colletotrichum Gloeosporioides. Postharvest Biol. Technol..

[14] García-Bramasco C.A., Blancas-Benitez F.J., Montaño-Leyva B., Medrano-Castellón L.M., Gutierrez-Martinez P., González-Estrada R.R. (2022). Influence of Marine Yeast Debaryomyces Hansenii on Antifungal and Physicochemical Properties of Chitosan-Based Films. J. Fungi.

[15] Shi Z., Wang F., Lu Y., Deng J. (2018). Combination of Chitosan and Salicylic Acid to Control Postharvest Green Mold Caused by Penicillium Digitatum in Grapefruit Fruit. Sci. Hortic..

[16] Waewthongrak W., Pisuchpen S., Leelasuphakul W. (2015). Effect of *Bacillus Subtilis* and Chitosan Applications on Green Mold (*Penicilium Digitatum* Sacc.) Decay in Citrus Fruit. Postharvest Biol. Technol..

[17] Shao X., Cao B., Xu F., Xie S., Yu D., Wang H. (2015). Effect of Postharvest Application of Chitosan Combined with Clove Oil against Citrus Green Mold. Postharvest Biol. Technol..

[18] Da Silva Y.C.R., Alves R.M., Da Silva B.M.P., Bron I.U., Cia P. (2020). Chitosan and Hot Water Treatments Reduce Postharvest Green Mould in ‘Murcott’ Tangor. J. Phytopathol..

[19] Correia J.H., Rodrigues J.A., Pimenta S., Dong T., Yang Z. (2021). Photodynamic Therapy Review: Principles, Photosensitizers, Applications, and Future Directions. Pharmaceutics.

[20] Wu X., Hu Y. (2022). Photodynamic Therapy for the Treatment of Fungal Infections. Infect. Drug Resist..

[21] Seididamyeh M., Netzel M.E., Mereddy R., Harmer J.R., Sultanbawa Y. (2023). Photodynamic Inactivation of Botrytis Cinerea Spores by Curcumin—Effect of Treatment Factors and Characterization of Photo-Generated Reactive Oxygen Species. Food Bioprocess Technol..

[22] Song L., Zhang F., Yu J., Wei C., Han Q., Meng X. (2020). Antifungal Effect and Possible Mechanism of Curcumin Mediated Photodynamic Technology against Penicillium Expansum. Postharvest Biol. Technol..

[23] Olmedo G.M., Cerioni L., González M.M., Cabrerizo F.M., Volentini S.I., Rapisarda V.A. (2017). UVA Photoactivation of Harmol Enhances Its Antifungal Activity against the Phytopathogens Penicillium Digitatum and Botrytis Cinerea. Front. Microbiol..

[24] Li X., Sheng L., Sbodio A.O., Zhang Z., Sun G., Blanco-Ulate B., Wang L. (2022). Photodynamic Control of Fungicide-Resistant Penicillium Digitatum by Vitamin K3 Water-Soluble Analogue. Food Control.

[25] Dibona-Villanueva L., Fuentealba D. (2021). Novel Chitosan-Riboflavin Conjugate with Visible Light-Enhanced Antifungal Properties against Penicillium Digitatum. J. Agric. Food Chem..

[26] Jiménez Jiménez B. (2021). Evaluación de un Biofungicida Fotoactivo en Hongos Patógenos de Frutales. Master’s Thesis.

[27] Pérez-Alfonso C.O., Martínez-Romero D., Zapata P.J., Serrano M., Valero D., Castillo S. (2012). The Effects of Essential Oils Carvacrol and Thymol on Growth of Penicillium Digitatum and P. Italicum Involved in Lemon Decay. Int. J. Food Microbiol..

[28] Bhatta U.K. (2022). Alternative Management Approaches of Citrus Diseases Caused by Penicillium Digitatum (Green Mold) and Penicillium Italicum (Blue Mold). Front. Plant Sci..

[29] Khalil Bagy H.M.M., Ibtesam B.F.M., Abou-Zaid E.A.A., Sabah B.M., Nashwa S.M.A. (2021). Control of Green Mold Disease Using Chitosan and Its Effect on Orange Properties during Cold Storage. Arch. Phytopathol. Plant Prot..

[30] Strano M.C., Altieri G., Admane N., Genovese F., Di Renzo G.C., Gill H., Garg H. (2017). Advance in Citrus Postharvest Management: Diseases, Cold Storage and Quality Evaluation. Citrus Pathology.

[31] Crocker L.B., Lee J.H., Mital S., Mills G.C., Schack S., Bistrović-Popov A., Franck C.O., Mela I., Kaminski C.F., Christie G. (2022). Tuning Riboflavin Derivatives for Photodynamic Inactivation of Pathogens. Sci. Rep..

[32] Piksa M., Lian C., Samuel I.C., Pawlik K.J., Samuel I.D.W., Matczyszyn K. (2023). The Role of the Light Source in Antimicrobial Photodynamic Therapy. Chem. Soc. Rev..

[33] Maisch T., Baier J., Franz B., Maier M., Landthaler M., Szeimies R.-M., Bäumler W. (2007). The Role of Singlet Oxygen and Oxygen Concentration in Photodynamic Inactivation of Bacteria. Proc. Natl. Acad. Sci. USA.

[34] Jori G., Magaraggia M., Fabris C., Soncin M., Camerin M., Tallandini L., Coppellotti O., Guidolin L. (2011). Photodynamic Inactivation of Microbial Pathogens: Disinfection of Water and Prevention of Water-Borne Diseases. J. Environ. Pathol. Toxicol. Oncol..

[35] Böcking T., Barrow K.D., Netting A.G., Chilcott T.C., Coster H.G.L., Höfer M. (2000). Effects of Singlet Oxygen on Membrane Sterols in the Yeast *Saccharomyces cerevisiae*. Eur. J. Biochem..

[36] Dibona-Villanueva L., Fuentealba D. (2022). Protoporphyrin IX–Chitosan Oligosaccharide Conjugate with Potent Antifungal Photodynamic Activity. J. Agric. Food Chem..

[37] Dai T., Fuchs B.B., Coleman J.J., Prates R.A., Astrakas C., St. Denis T.G., Ribeiro M.S., Mylonakis E., Hamblin M.R., Tegos G.P. (2012). Concepts and Principles of Photodynamic Therapy as an Alternative Antifungal Discovery Platform. Front. Microbiol..

