# Supplementary material for: Effect of Chitosan–Riboflavin Bioconjugate on Green Mold Caused by Penicillium digitatum in Lemon Fruit
Source: Polymers (Basel). 2024 Mar 23;16(7):884. doi: 10.3390/polym16070884 (PMC11013941; doi:10.3390/polym16070884)
Supplement: Supplementary file 1 [file polymers-16-00884-s001.zip › polymers-2893323-supplementary.pdf]

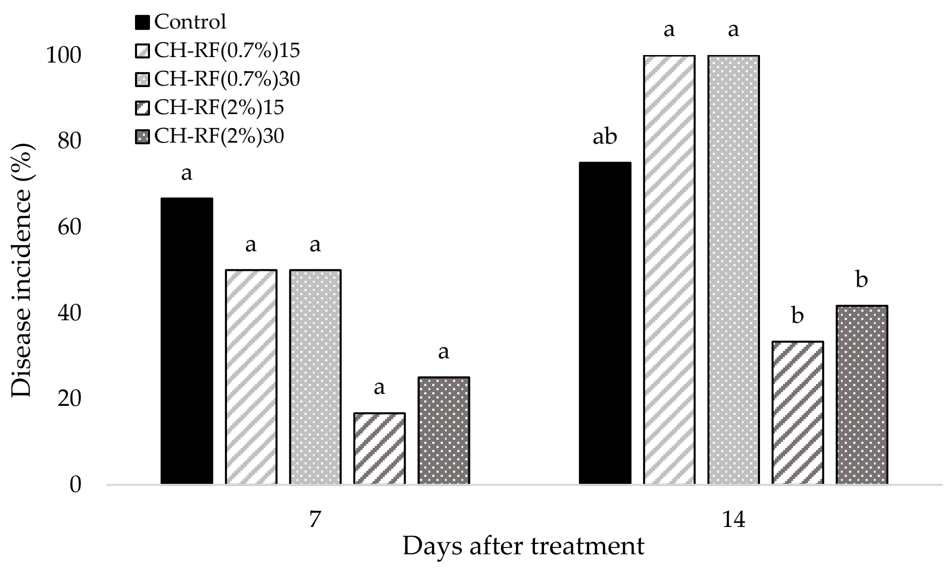

Figure S1. Disease incidence (%) of green mold on lemons inoculated and treated with sterile water (Control); Chitosan-Riboflavin conjugate at 0.7% with 15 (CH-RF0,7% 15) and 30 min (CH-RF0.7% 30) of LED exposure or CH-RF at 2% with 15 (CH-RF2% 15) and 30 min (CH-RF2% 30) of LED. The lemons were evaluated after 7 and 14 days under temperate and humid conditions (20°C±1°C; 90-95%RH). Different letters indicate significant statistical differences of the means by Tukey's test.

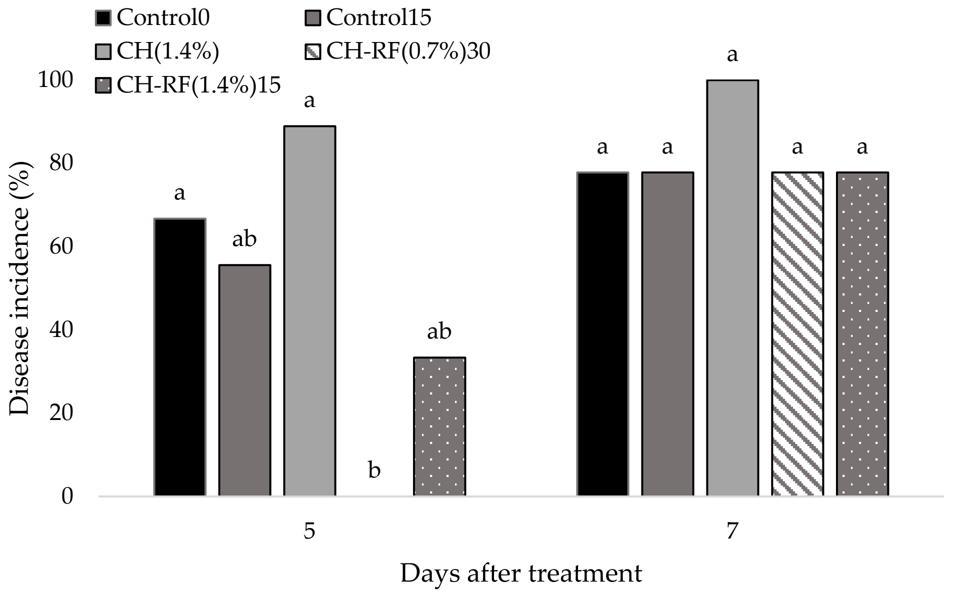

Figure S2. Disease incidence (%) of green mold on lemons inoculated and treated with sterile water without (Control0) and with 15 min of white LED exposure (Control15); Chitosan at 1.4% (CH1.4%); Chitosan-Riboflavin conjugate at 0.7% with 30 min of LED exposure (CH-RF0.7% 30) and CH-RF at 1.4% with 15 min of LED exposure (CH-RF1.4% 15). The lemons were evaluated after 5 and 7 days under temperate and humid conditions (20°C±1°C; 90-95%RH). Different letters indicate significant statistical differences of the means by Tukey's test.

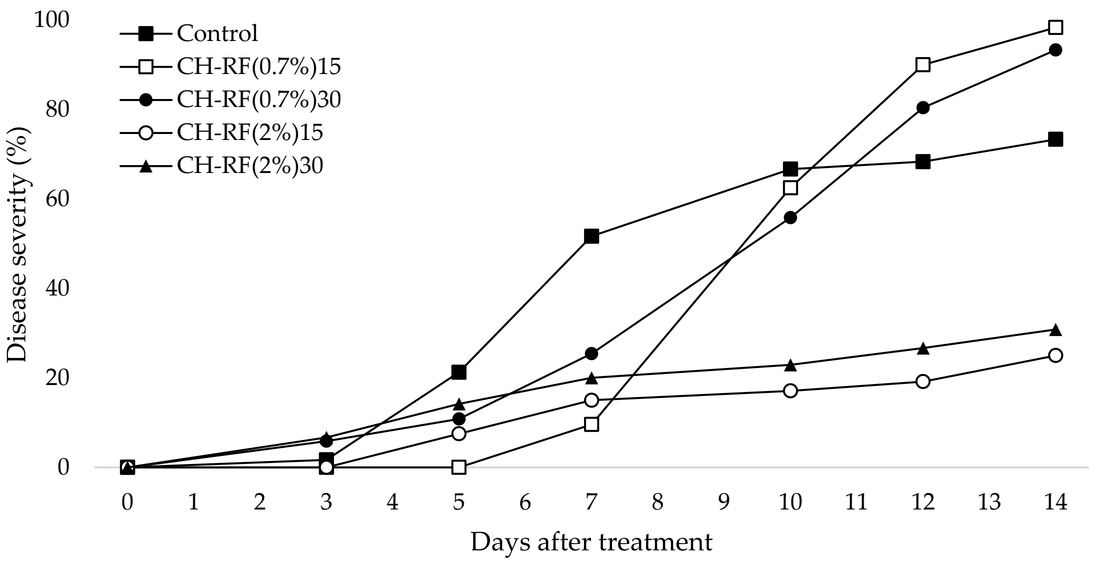

Figure S3. Disease severity (%) of green mold on lemons inoculated and treated with sterile water (Control); Chitosan-Riboflavin conjugate at 0.7% with 15 (CH-RF0.7% 15) and 30 min (CH-RF0,7% 30) of LED exposure or CH-RF at 2% with 15 (CH-RF2% 15) and 30 min (CH-RF2% 30) of LED. The lemons were evaluated after 3, 5, 7,10, 12 and 14 days under temperate and humid conditions (20°C±1°C; 90-95%RH).

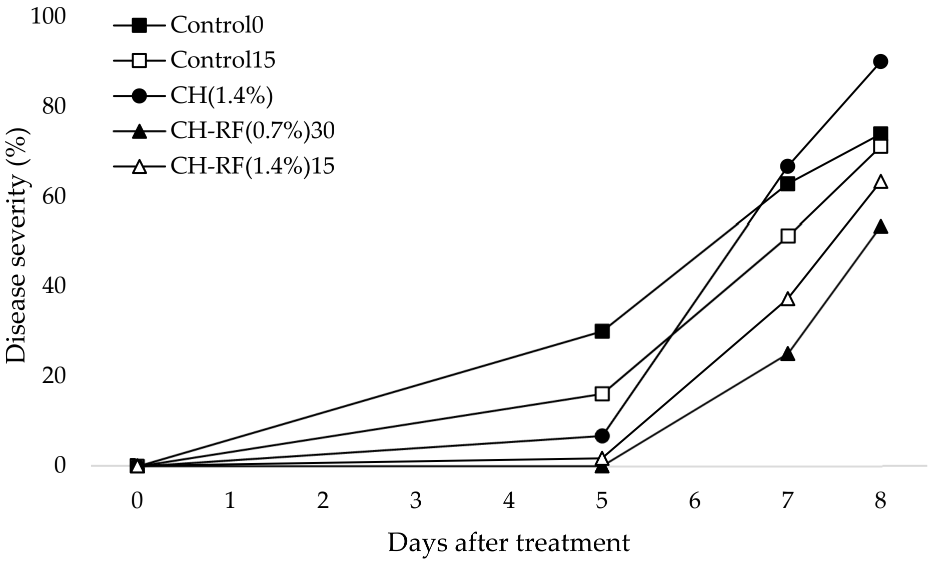

Figure S4. Disease severity (%) of green mold on lemons inoculated and treated with sterile water without (Control0) and with 15 min of white LED exposure (Control15); Chitosan at 1.4% (CH1.4%); Chitosan-Riboflavin conjugate at 0.7% with 30 min of LED exposure (CH-RF0.7% 30) and CH-RF at 1.4% with 15 min of LED exposure (CH-RF1.4% 15). The lemons were evaluated after 5, 7 and 8 days under temperate and humid conditions (20°C±1°C; 90-95%RH).
